# Effects of a Telerehabilitation Program in Women with Fibromyalgia at 6-Month Follow-Up: Secondary Analysis of a Randomized Clinical Trial

**DOI:** 10.3390/biomedicines10123024

**Published:** 2022-11-23

**Authors:** Ignacio Hernando-Garijo, Ricardo Medrano-de-la-Fuente, Sandra Jiménez-del-Barrio, María Teresa Mingo-Gómez, Héctor Hernández-Lázaro, Silvia Lahuerta-Martin, Luis Ceballos-Laita

**Affiliations:** 1 Faculty of Health Sciences, University of Valladolid, 47005 Valladolid, Soria, Spain; 2 Clinical Research in Health Sciences Group, University of Valladolid, 47005 Valladolid, Soria, Spain; 3 Ólvega Primary Care Health Center (Soria, Spain), Soria Health Care Management, Castilla y León Regional Health Management (SACYL), 47005 Valladolid, Spain; 4 Faculty of Health Sciences, University of Zaragoza, 50009 Zaragoza, Aragon, Spain

**Keywords:** chronic pain, exercise, fibromyalgia, telemedicine, telerehabilitation

## Abstract

(1) Background: Telerehabilitation allows health professionals to monitor patients without face-to-face contact. The objective was to analyze the effects of a telerehabilitation program based on aerobic exercise in women with fibromyalgia at 6-month follow-up. (2) Methods: Participants were randomized into the telerehabilitation group (*n* = 17) or the control group (*n* = 17). The telerehabilitation group performed 30 sessions of exercise for 15 weeks. The exercises were guided by video and adjusted by videocalls. Pain intensity, fibromyalgia impact, physical function, isometric strength and quality of life were measured at baseline and at 6 months after the end of the intervention. (3) Results: There were no between-group differences in pain intensity, fibromyalgia impact, physical function, isometric strength or quality of life at 6-month follow-up (*p* > 0.05). (4) Conclusion: A telerehabilitation exercise program based on aerobic exercises may not be an effective treatment for women with fibromyalgia at 6 months of follow-up due to the lack of between-group differences in any variable.

## 1. Introduction

Fibromyalgia (FM) is a chronic syndrome with a worldwide prevalence of 2.1%. FM most frequently affects middle-aged women [1]. The aetiology of FM is complex and remains unclear; however, a sensitization of the central nervous system has been suggested [2]. This syndrome is characterized by the presence of persistent generalized pain that is associated with other symptoms such as weakness and impaired physical function [3,4]. Consequently, FM implies an impact on the quality of life of patients with FM and significant socioeconomic and health-related costs [1].

Due to the clinical an etiological complexity of this pathology, a wide range of interventions have been described. Currently, the main clinical guidelines indicate that non-pharmacological conservative therapies should be the basis of treatment [5,6]. Therapeutic exercise programs are the principal interventions. These programs have shown positive effects on pain intensity and physical function in the short- and medium- term [6,7]. Among the different exercise programs, aerobic exercise interventions appear to be the most accepted for patients with FM. However, there is a lack of evidence of their effects in the long term [5,6,7].

The lockdown and restrictions to prevent the spread of COVID-19 have resulted in negative effects by limiting non-pharmacological conservative treatments in patients with chronic diseases such as FM [8]. For this reason, the design of therapeutic interventions without the risk of viral infection has become necessary to improve the functional capacity of and decrease symptoms in patients with FM [9]. Telerehabilitation (TR) has been proposed as a feasible method to guide certain therapies such as therapeutic exercise and maintain social distancing while maintaining therapist–patient communication [10]. Therapeutic exercise applied with TR allows therapists to establish a specific intervention and to promote self-management. Although short-term benefits have been identified [11], the long-term effects of therapeutic exercise applied through telematic strategies in patients with FM have not been analyzed.

For this reason, the aim of this study was to analyze the effects of a TR program based on aerobic exercise during the 15-week Spanish home lockdown in patients with FM at 6 months of follow-up.

## 2. Materials and Methods

### 2.1. Study Design

A single-blind randomized clinical trial was conducted between May 2020 and January 2021 according to the Consolidated Standards of Reporting Trials (CONSORT) Guidelines [12]. This study was registered at clinicaltrials.gov (accessed on 1 September 2022) with the registration number NCT04340674. Ethical approval was obtained from the Research Ethics Committee of Valladolid West Health Area (CASVE-NM-19-410). Helsinki and Taipei declarations were considered for the study. All participants signed an informed consent form prior to participation.

### 2.2. Participants

Patients were recruited from the Rheumatology service of the Soria Healthcare Complex from February to March 2020.

The inclusion criteria were women diagnosed with FM by a rheumatologist according to the American College of Rheumatology criteria from 2016 [3], aged between 30 and 75 years with internet access from any type of digital device.

The exclusion criteria were the presence of medical contraindications for exercise, systemic diseases, somatic or psychiatric disorders, pregnancy or breastfeeding, exposure to other physical therapy intervention or modifications in pharmacological treatment during the study or in the previous three months.

### 2.3. Sample Size

The sample size calculation was performed using the Minitab^®^ 13.0 program and considered pain intensity as the primary outcome. A value of 2 cm in the visual analogue scale (VAS) was considered as the minimum clinically important difference (MCID) for chronic pain patients [13]. The standard deviation was based on a pilot study in patients with FM. A two-tail test with a confidence interval (CI) of 95%, a statistical power of 80% and a drop-up of 15% was established. Assuming a between-group mean difference of 2 cm and a standard deviation of 1.9 cm, 34 participants were necessary for both groups.

### 2.4. Randomization

Participants who met the inclusion criteria were randomly assigned to both groups: the TR group or the control group. Randomization (1:1 ratio) was performed by an independent researcher who did not participate in the data collection or the statistical analysis using GraphPad QuickCalcs (GraphPad Software^®^, San Diego, CA, USA).

### 2.5. Intervention

The TR group received a protocol based on aerobic exercise during the 15 weeks. The control group received no additional intervention to the usual pharmacological treatment. Both groups were instructed to keep their medication stable during the study. A physical therapist experienced in treating patients with chronic pain using therapeutic exercise carried out the intervention.

The TR group performed 30 sessions of aerobic exercise over a period of 15 weeks. The protocol was based on rhythmic, low-impact exercises guided by video. Participants performed moderate intensity exercises with a frequency of two sessions per week. Each session lasted 50 min and included three parts: warm-up, aerobic exercise and cool down. The Borg CR-10 scale was used to adjust the exercise intensity in each session according to the participant’s effort [14]. Details of the exercise training protocol were described in a previous study [11].

Participants in both groups were contacted by video call once a week. The participants of the TR group were called to adjust the exercises. Video calls in the control group were used to ensure that the initial conditions did not change during the study.

### 2.6. Outcome Measures

Two physical therapists blinded to the group allocation performed the measurements. At baseline, clinical and demographic data were collected for the entire sample, including age, gender, weight, body mass index (BMI) and drug intake. Primary and secondary outcomes were collected at baseline and at 6 months after the intervention. 

The primary outcome was pain intensity, which was assessed using VAS. This scale consists of a 10-cm horizontal line in which 0 represented “no pain” and 10 represented “the most intense pain imaginable”. The VAS has shown excellent reliability, with an intraclass correlation coefficient (ICC) of 0.99 [15].

#### Secondary Outcomes

FM impact was assessed using the Spanish version of the Revised Fibromyalgia Impact Questionnaire (FIQ-R). This questionnaire consists of 21 items and the score ranging from 0 to 100 points, in which higher scores indicate a greater FM impact. The FIQ-R has shown excellent reliability (CCI = 0.81) [16].

Physical function was assessed with a test battery used in patients with FM which has shown excellent reliability (CCI = 0.80–0.98). Each test was performed twice, and the mean was recorded [17]:

- Chair Stand Test (30-s CST): the number of times that participants were able to get up and sit on a chair without armrests within 30 s was measured.
- 6-Minute Walk Test (6MWT): the distance that the participants could walk in 6 min along a corridor of 20 m was measured.
- Arm Curl Test (ACT): the number of flexion–extension elbow movements that participants were able to perform holding a 2.3 kg weight with the dominant arm within 30 s was recorded.
- Timed Up and Go (TUG): the time that the participants took to stand up from a chair, walk 3 m, return and sit down again was recorded.

Elbow and knee isometric strength was assessed using Lafayette Instrument^®^ model 01165 manual dynamometer. For elbow strength, participants sat on a chair, with 90° elbows flexion. The dynamometer was placed on the anterior part of the distal forearm region. Participants were asked to perform maximum isometric elbow flexion. This procedure has shown good–excellent reliability (ICC = 0.74–0.95) [18]. For knee strength, participants sat on a chair, with 90° knee flexion. The dynamometer was placed on the anterior part of the tibia. Participants were asked to perform maximal isometric knee extension (ICC = 0.77–0.95) [19]. The maximum score of three measurements was recorded in both tests.

The quality of life was assessed using the Spanish version of the Health Assessment Questionnaire (HAQ). It consists of 20 items with a final score between 0 and 3 points, in which higher values represent lower quality of life. The HAQ has shown excellent reliability (ICC = 0.89) [20].

### 2.7. Statistical Analysis

A statistical analysis was performed using the Statistical Package for Social Sciences^®^ (SPSS), version 24.0 for Windows. An intention-to-treat analysis was performed. Means and standard deviations were calculated for quantitative variables. Frequencies and percentages were estimated for qualitative variables. The Shapiro–Wilk test was used to assess the normal distribution for quantitative data. Comparisons between groups were analyzed by Student´s *t*-test or the Mann–Whitney U test, for normally distributed data or non-normally distributed data, respectively. The Chi-square test (X^2^ test) was used to compare the nominal variables. The Group × Time interaction between both groups (TR group and control group) and time points (baseline and 6-month follow-up) was performed using two-way analysis of variance (ANOVA). A *p*-value < 0.05 was considered statistically significant. The effect size (Cohen’s d) was also calculated. The magnitude of difference was classified as small if the value of Cohen’s d ranged from 0.2 to 0.5, as moderate if it ranged from 0.5 to 0.8 or as large if it was greater than 0.8.

## 3. Results

**Figure 1:**
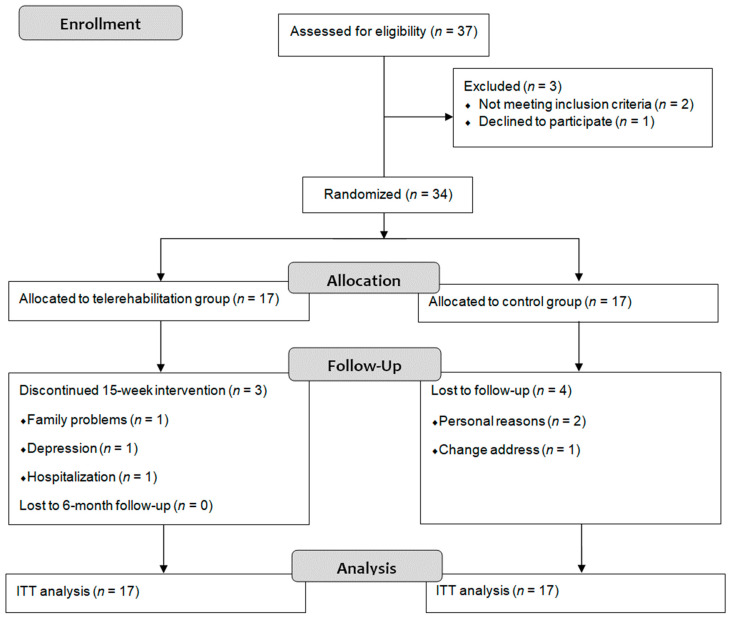
Flow diagram. ITT: intention-to-treat.

Thirty-seven women with an FM diagnosis were referred from the rheumatology service, and finally 34 were included. Two patients did not meet the selection criteria and one patient refused to participate for personal reasons. The participants were assigned to the TR group (*n* = 17) or to the control group (*n* = 17). Six participants dropped out of the study during the intervention period and one more during the follow-up: three in the TR group and four in the control group. The flowchart is shown in Figure 1.

No statistically significant between-group differences (*p* > 0.05) were found at the beginning of the study in any demographic or clinical variable. The sociodemographic characteristics and medication intakes at baseline are shown in Table 1.

No statistically significant differences were identified between both groups in any outcome at 6 months of follow-up (*p* > 0.05). Regarding within-group analysis in the TR group, there was a statistically significant decrease in pain intensity (*p* = 0.034; d = 0.7) and FM impact (*p* = 0.010; d = 0.7), an improvement in physical function through 30 s-CST (*p* = 0.006; d = 0.6), 6MWT (*p* < 0.001; d = 0.6), ACT (*p* = 0.039; d = 0.5) and TUG (*p* = 0.042; d = 0.6), an increase in the isometric strength of the right (*p* = 0.035; d = 0.5) and the left (*p* = 0.036; d = 0.6) elbow and the right (*p* = 0.002; d = 0.7) and the left (*p* = 0.002; d = 0.9) knee and an improvement in quality of life (*p* = 0.015; d = 0.5) at 6-month follow-up. The effect sizes in the TR group were moderate to large. In the control group, there were no differences (*p* > 0.05; d < 0.5) between baseline and follow-up in any outcome, and the effect sizes were small. Within-group and between-group changes and effect sizes for primary and secondary outcomes are shown in Table 2.

## 4. Discussion

The present study analyzed the effects of a TR approach in patients with FM at 6-month follow-up after a 15-week exercise intervention. This is the first study to analyze the long-term effects of an exercise program through non-face-to-face strategies on physical function and strength in patients with FM.

Regarding pain intensity, no between-groups differences were found at 6 months of follow-up. Several studies have assessed the immediate and medium-term effects of home-based interventions, but the long-term effects of TR approaches remained unknown. Previous studies found a short-term decrease in pain intensity after TR-based aerobic exercise [11]. Other home-based interventions with a frequency of two–three exercise sessions per week have shown medium or long-term effects on pain intensity [21,22]. Da Costa et al. [21] concluded that a 12-week exercise program was more effective than a control group in reducing pain in the long-term, but only in the upper body. The within-group results of Evcik et al. [22] showed a medium-term reduction in pain through a 5-week exercise program, but these effects were not maintained in the long term. The results of these studies are similar to the results achieved in our study, which suggests that proposing prolonged exercise programs could be key to achieving sustained benefits. In this sense, the majority of patients with FM are sedentary and could require a long intervention to adopt active habits [4,23]. A meta-analysis suggested that aerobic exercise could promote longer-lasting effects than other types of exercise, although the most appropriate intensity and frequency of exercise sessions have not been specified [7].

The results on FM impact did not show between-groups differences at 6-month follow-up, although this variable was statistically significantly reduced within the TR group. Previous studies showed conflictive results on FM impact [21,24]. Da Costa et al. [21] found no between-groups differences between a home-based exercise and a control group in the FM impact in patients with FM after the intervention and at 3 months follow-up. However, Mannerkorpi et al. [24] found benefits in terms of the FM impact after face-to-face intervention. This could mean that face-to-face interventions are more effective in reducing the impact of FM due to exercise management and/or psychosocial reasons derived from face-to-face interventions.

No statistically significant differences were identified between both groups in terms of strength and physical function. Other studies identified certain benefits in terms of strength and physical function after aerobic exercise interventions [24,25]. Despite this, it is important to note that those studies presented some differences. According to Jones et al. [26], all the patients included in our study were worse than the fitness standards for older adults <64 years with FM. The mean values presented in the 6MWT by the patients included in the study by Mannerkorpi et al. [24] were higher than 600 in select patients, which could mean that select patients presented no disability. In addition, the patients had a low attendance rate, with a median attendance rate of 62% and 50% in the experimental and control group, respectively. Thus, the positive effects concluded by the authors are unclear. It is important to note that the objective of aerobic exercise is to improve cardiorespiratory fitness. Therefore, the lack of improvement in a strength test could be because the volume and intensity of the exercises were designed for aerobic training and not for resistance training. The results achieved in the physical function test could be related to the lack of a decrease in pain intensity. A reduction in pain intensity can produce an improvement in muscle function and cognitive ability in patients with FM [27,28]. Therefore, since the pain did not decrease, the physical function could not improve.

Concerning quality of life, an improvement in quality of life was observed within the TR group 6 months after the intervention, which was not enough to reach between-group differences. Certain authors have established a relationship between the quality of life and physical function in patients with FM [29]. Thus, the lack of improvement in quality of life could be conditioned by the lack of improvement in physical function.

FM is a chronic condition with fluctuating symptoms and periodical exacerbations. In this sense, a TR program based on aerobic exercise failed to achieve statistically significant improvements compared to a control group. Despite of certain authors concluding a positive effect in terms of the aerobic training, it is necessary to consider several factors, such as the attendance rate and the mean values for symptoms and physical function, that the sample presented at the beginning of the study.

We have identified three main limitations in this study. First, the sample was exclusively composed of women, so it was not fully representative of the FM population. Second, the small sample size could have prevented the detection of between-group differences for specific outcome variables. Finally, no additional intervention was added to the control group.

## 5. Conclusions

The results of this study demonstrate that a TR-based approach on exercise offered a similar therapeutic effect compared to the control group in all outcome measures at 6 months in women with FM.

## Figures and Tables

**Table 1:** Sociodemographic characteristics and medication intakes at baseline.

	TR Group Mean ± SD	Control Group Mean ± SD	Significance
Age (years)	51.81 ± 9.05	55.06 ± 8.51	0.304 ^a^
Height (cm)	158 ± 6.29	161.81 ± 5.13	0.191 ^b^
Weight (kg)	68.19 ± 16.88	68.13 ± 15.10	0.836 ^b^
BMI (kg/cm^2^)	27.25 ± 7.30	25.93 ± 5.27	0.598 ^b^
Medication n (%)	17 (100)	17 (100)	0.224 ^c^
Anxiolytics n (%)	9 (53)	7 (41)	
Antidepressants n (%)	9 (53)	9 (53)	
Anti-inflammatory n (%)	14 (82)	8 (47)	
Analgesics n (%)	11 (65)	12 (71)	
Muscle relaxants n (%)	4 (24)	2 (12)	

^a^: t Student; ^b^: U Mann–Whitney; ^c^: Chi-square; TR: telerehabilitation; BMI: body mass index. Mean and standard deviation (SD) for quantitative data. Percentages (%) and frequencies (n) for qualitative data.

**Table 2:** Within-group changes, between-group changes and effect sizes for primary and secondary outcomes at baseline and 6-month follow-up.

	Baseline Mean ± SD	6-Month Follow-Up Mean ± SD	Within-Group Changes (*p* Value)	Effect Size (Cohen d)	Between-Group Changes (*p* Value)	Between-Group Effect Size (Cohen d)
**VAS** (**0–10**)						
TR group	7.08 ± 1.45	6.15 ± 1.25	0.93 (0.08;1.79) 0.034	0.7	F = 0.02 0.873	0.2
Control group	7.29 ± 1.07	6.52 ± 2.06	0.77 (−0.48; 2.03) 0.205	0.4		
**FIQ**-**R**						
TR group	59.44 ± 9.04	53.26 ± 9.40	6.18 (1.70; 10.44) 0.010	0.7	F = 0.25 0.612	0.2
Control group	55.36 ± 16.46	50.34 ± 19.45	5.01 (−4.46; 15.08) 0.261	0.2		
**30s**-**CST**						
TR group	7.46 ± 2.66	8.92 ± 2.46	−1.46 (−2.40; −0.51) 0.006	0.6	F = 0.46 0.494	0.3
Control group	7.14 ± 4.20	8.00 ± 4.31	−0.85 (−2.03; 0.31) 0.139	0.2		
**6MWT**						
TR group	403.57 ± 107.13	458.93 ± 67.40	−55.36 (−22.13; −85.50) <0.001	0.6	F = 2.29 0.115	0.6
Control group	407.01 ± 137.09	374.94 ± 173.33	32.07 (−14.78; 61.22) 0.316	0.2		
**ACT**						
TR group	9.60 ± 4.31	11.61 ± 3.12	−2.23 (−4.32; −0.13) 0.039	0.5	F = 0.95 0.338	0.4
Control group	10.06 ± 5.56	10.00 ± 5.14	−0.05 (−1.77; 0.77) 0.413	0.0		
**TUG**						
TR group	9.19 ± 2.98	7.72 ± 1.17	1.47 (0.05; 2.88) 0.042	0.6	F =2.52 0.109	0.7
Control group	10.69 ± 4.73	10.26 ± 5.15	0.43 (−1.63; 2.49) 0.657	0.1		
**Right elbow strength**						
TR group	7.36 ± 4.14	9.29 ± 4.21	−1.92 (−3.69; −0.16) 0.035	0.5	F = 0.75 0.393	0.3
Control group	7.85 ± 3.31	7.95 ± 3.75	−0.10 (−1.28; 1.08) 0.857	0.0		
**Left elbow strength**						
TR group	7.56 ± 3.34	9.49 ± 3.55	−1.92 (−3.70; −0.14) 0.036	0.6	F = 1.06 0.312	0.4
Control group	8.40 ± 3.58	8.06 ± 3.65	0.34 (−1.54; 2.23) 0.697	0.1		
**Right knee strength**						
TR group	7.86 ± 3.39	9.96 ± 2.69	−2.10 (−3.28; −0.91) 0.002	0.7	F = 0.04 0.845	0.1
Control group	9.13 ± 4.22	9.66 ± 4.69	−0.53 (−2.47; 1.40) 0.557	0.1		
**Left knee strength**						
TR group	7.14 ± 2.68	9.78 ± 3.02	−2.64 (−3.28; −0.91) 0.002	0.9	F = 0.33 0.580	0.2
Control group	9.30 ± 4.53	8.95 ± 4.26	0.34 (−1.06; 1.74) 0.604	0.1		
**HAQ**						
TR group	1.64 ± 0.63	1.31 ± 0.61	0.32 (0.07; 0.57) 0.015	0.5	0.871	0.0
Control group	1.48 ± 0.56	1.30 ± 0.72	0.18 (−0.05; 0.49) 0.108	0.2		

SD: standard deviation; TR: telerehabilitation; VAS: visual analogue scale; FIQ-R: Revised Fibromyalgia Impact Questionnaire; 30s-CST: Chair Stand Test; 6MWT: 6-Minute Walk Test; ACT: Arm Curl Test; TUG: Timed Up and Go; HAQ: Health Assessment Questionnaire.

## Data Availability

The anonymized data are available from the corresponding author upon a reasonable request.
